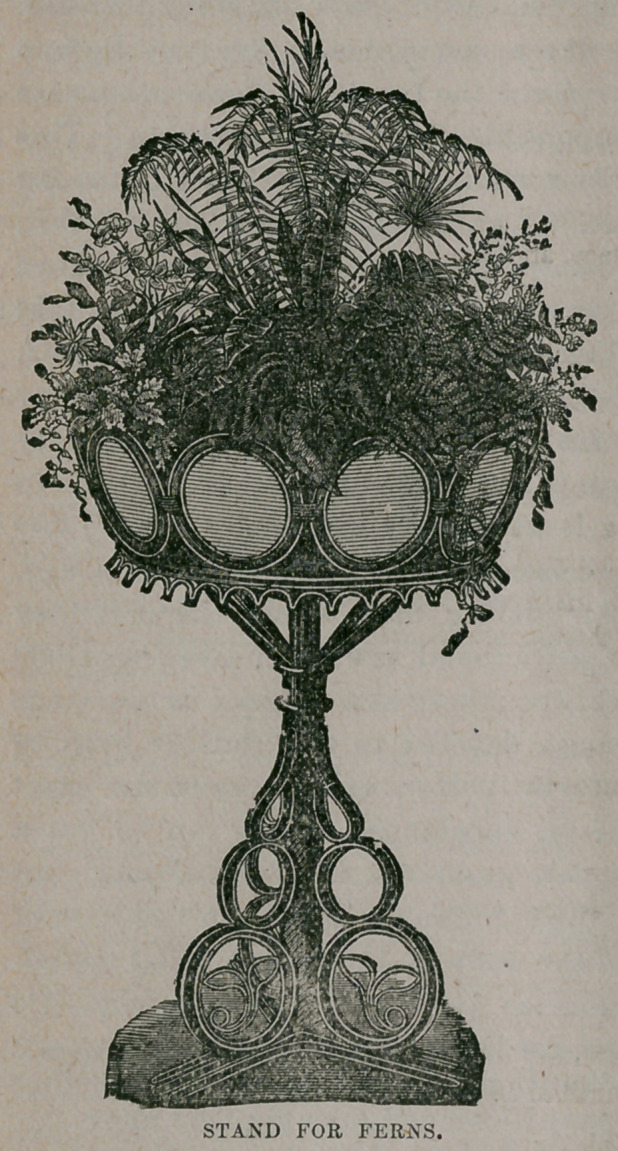# Household

**Published:** 1888-12

**Authors:** 


### HOUSEHOLD.

A Fern Paradise at Home.—It is not only the poor who have to live in gardenless dwellings and look out from sunless windows. The mansions of the rich, and thousands of houses of the well-to-do and of the middle classes are necessarily in great cities placed where the sun cannot exert his charming life-giving influence. Many a window of a grand house looks out upon nothing but brick walls, which tower up high and blot out the sun's rays. The occupants of these houses are often bound by the exigencies of business to make their homes for weary months in these shadowy dwelling places. Why then do they not bring the beautiful ferns into requisition ?

STAND FOR FERNS.

What exquisite grace would be shed over every room in a house if every available space were occupied by the feathery fronds of those beautiful plants ! On tables and side-boards, on mantel pieces and on window sills ; hanging from window rods, on the landing of the stairs, in the hall, in the bedroom—everywhere in fact.

We give an illustration of a cane stand lined with zinc and filled with ferns and begonias, which might be bought for a dollar or two, and which would not be out of place in the most elegant apartments.

Uses of Hard and Soft Water in Cooking.—All cooks understand the different effects produced by soft and hard water in cooking meat and vegetables. Peas and beans cooked in hard water containing lime or gypsum, will not boil tender, because these substances harden vegetable caseine. Many vegetables, as onions, boil nearly tasteless in soft water, because all the flavor is boiled out. The addition of salt often checks this, as in the case of onions, causing the vegetables to retain the peculiar flavoring principles, besides such nutritious matter as might be lost in soft water. For extracting the juice of meat to make a broth or soup, soft water, unsalted and cold at first, is best, for it much more readily penetrates the tissues ; but for boiling where the juice should be retained, hard water or soft water salted is preferable, and the meat should be put in while the water is boiling, so as to seal up the pours at once.

## Figures and Tables

**Figure f1:**